# Mineral homeostasis in late preterm infants

**DOI:** 10.1186/1824-7288-40-S2-A15

**Published:** 2014-10-09

**Authors:** Paolo Ghirri, Sara Lunardi, Livia Leuzzi, Giampiero I Baroncelli, Francesca Moscuzza, Antonio Boldrini

**Affiliations:** 1 Division of Neonatology and Neonatal Intensive Care Unit, Hospital Santa Chiara, Pisa, Italy; 2 Pediatric Clinic, University Hospital of Pisa, Italy

## 

80% of mineral accretion take place in the third trimester of gestation with a fetal accretion rates of 100-150 mg/kg/day for calcium, 50-70 mg/kg/day for phosphate and 3 mg/kg/day for magnesium. During the third trimester of gestation fetal weight triples but Ca content quadruples with a faster increase in fetal body Ca accretion from 32 to 40 weeks of gestation [1]. As a result, late preterm infants will be deprived of the intrauterine supply of calcium and phosphorus during a period of rapid skeletal growth affecting bone mineral mass. Late preterm infants have a higher incidence of hypocalcemia [2] and represent an intermediate risk category as compared with term and very preterm infants. AGA late-preterm infants have a higher bone turn-over than term infants, but smaller than very pre-term newborns [3]. During the third trimester of gestation, bone mineral density (BMD) increases at a faster rate in utero (term infants) than ex utero (preterm infant) according to gestational age. At term newborns have a physiological reduction in BMD in the first 2-3 months of life with a recovery during the first year of life. Preterm newborns present a similar event with a higher reduction of mineral retention from birth to term in the presence of high skeletal growth. Inadequate supply of calcium and phosphate to newborns requiring parental nutrition and several drugs (steroids, methylxanthines, diuretics) may increase the risk of osteopenia. Drugs affect bone metabolism decreasing calcium absorption and osteoblasts proliferation and increasing calcium renal excretion and osteoclasts activation. How do we best support the rapid skeletal growth of late preterm infants? Have early nutrition during the first weeks of life been adequate? Enriched formulas for preterm newborns have a positive and probably long-term effect on bone mineralization [4,5]. In late preterm infants the goal should be to provide nutrients based on gestational age instead of relying on birthweight. However there are no specific recommendation and further studies are warranted to determine the best care for late preterm infants.
